# Influence of the Compliance of a Technological System on the Machining Accuracy of Low-Stiffness Shafts in the Grinding Process

**DOI:** 10.3390/ma16041498

**Published:** 2023-02-10

**Authors:** Antoni Świć, Arkadiusz Gola

**Affiliations:** Department of Production Computerisation and Robotisation, Faculty of Mechanical Engineering, University of Technology, ul. Nadbystrzycka 36, 20-618 Lublin, Poland

**Keywords:** low-stiffness shaft, control, elastic-deformable state, grinding, compliance adjustment, technological methods, machining accuracy

## Abstract

This paper reports the results of research on the influence of the compliance of the technological system used in grinding low-stiffness shafts on the shape accuracy of the workpieces. The level of accuracy achieved using passive compliance compensation was assessed, and technological assumptions were formulated to further increase the shape accuracy of the low-stiffness shafts obtained in the grinding process. Taking into account the limitations of passive compliance compensation, a method for the active compensation of the compliance of the elastic technological system during the machining process was developed. The experiments showed that the accuracy of grinding was most effectively increased by adjusting the compliance and controlling the bending moments, depending on the position of the cutting force (grinding wheel) along the part. The experimental results were largely consistent with the results of the theoretical study and confirmed the assumptions made. Adjusting the compliance in the proposed way allows for the significant improvement in the accuracy and productivity of machining of low-stiffness shafts.

## 1. Introduction

The desire to reduce the amount of materials used in the construction of mechanisms and machines, as well as the functional uses of some parts, makes it necessary to produce precise low-stiffness parts [1,2,3,4,5,6]. Rotationally symmetrical parts (shafts, turbine rotors, pumps, lead screws, etc.) account for about 34% of all parts used in the engineering industry, of which 12% are classified as low-stiffness parts [7,8,9,10]. Such parts are characterized by disproportionate overall dimensions and low stiffness in specific directions and sections [5,11,12,13]. The traditional machining methods used in the production of such parts are inefficient and do not allow the obtaining of the required shape, dimensional accuracy, and surface quality [14,15,16,17]. The conventional approach is to use multi-pass machining with reduced values of cutting parameters, rests, and additional uneconomic and inefficient-lapping operations [18,19,20,21,22].

The low stiffness of such parts, which is lower than the stiffness of the machine tool, can (under certain conditions) cause vibrations and the occurrence of factors that disrupt and destabilize the machining process [23,24,25,26].

The errors in the longitudinal section of the workpiece that occur during machining are caused by the low and uneven stiffness of the technological system, the main elements of which are the workpiece, the centers in which it is mounted, and the cutting tool [27,28,29,30]. The compliance (flexibility) of such a system can be altered by changing the stiffness of the centers or increasing the stiffness of the part in the machining process, for example, as a result of applying the appropriately defined bending moments [31,32,33,34].

The accuracy of grinding of low-stiffness shafts can, therefore, be significantly increased by using technological machining methods that allow for the control of the compliance of the technological system, while the efficiency of the process can be significantly increased by using higher machining parameters [35,36,37].

## 2. Influence of the Compliance of the Technological System on the Accuracy of the Shape during Grinding

**Figure 1:**
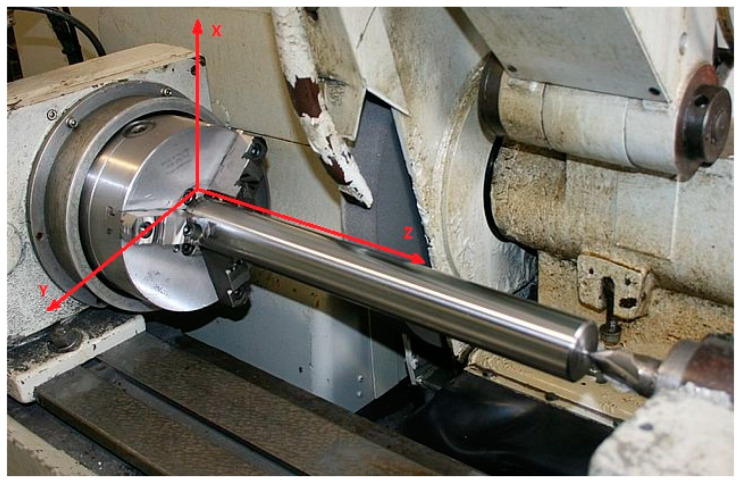
A schematic view of the grinding process with the defined coordinates.

The main cause of shape errors in the longitudinal section of a workpiece is the uneven stiffness of the technological system along the X-axis in the place where the cutting force is applied (Figure 1). A shape error of the machined surface can be reduced by equalizing the stiffness of the technological system in different cross-sections of the blank.

The relationship describing the surface of a workpiece made in one pass and intended for machining with the use of grinding operations can be given by [38]:

(1) $$y(x)=F_{p}\left[\frac{{\left(1-x/L\right)}^{2}}{j_{s}}+\frac{{\left(x/L\right)}^{2}}{j_{t}}+\frac{x^{2}\cdot {\left(1-x\right)}^{2}}{3\cdot E\cdot I\cdot L}+\frac{1}{j_{gw}}\right],$$

where:

*F_p_*—resistive (radial) cutting force component;
x—distance of the force application point from the end of the shaft;
*L*—length of the part;
*j_s_*, *j_t_*, *j_gw_—*stiffness of the spindle, tailstock, and grinding wheel, respectively;
*E*—Young’s modulus;
*I—*moment of the section inertia.

After the substitution *ε = x*/L, *K =* L/d, *E =* 2 × 10^11^ [N/m^2^]; *I = π* ∙ *d*^4^/64 *=* 0.05 ∙ *d*^4^, Equation (1) takes the following form [38]:

(2) $$y(x)=K_{r}f\left[\frac{{\left(1-\varepsilon \right)}^{2}}{j_{s}}+\frac{\varepsilon^{2}}{j_{t}}+\frac{\varepsilon^{2}\cdot {\left(1-\varepsilon \right)}^{2}\cdot K^{3}}{30\cdot d}+\frac{1}{j_{gw}}\right],$$

where:

*K_r_*—a coefficient that depends on the stiffness of the technological system, process parameters, and grinding conditions;
*f*—feed;
*d*—diameter of the part.

The relationship in square brackets in formula (2) describes the technological system’s compliance ω_c_(ε) in various cross-sections of the workpiece [38]. The non-uniformity of the compliance Δω_c_ is determined by the difference between maximum (ω_c(max)_) and minimum (ω_c(min)_) compliance values:

(3) $$\Delta \omega_{c}=\omega_{c(\max)}-\omega_{c(\min)},$$

The machining accuracy of low-stiffness shafts with a diameter larger than 50 mm can be increased by increasing the compliance of the headstock and tailstock to the following values [38]:

(4) $$\omega_{w,k}=4\cdot 10^{-12}\frac{K^{3}}{d}$$

As the total compliance increases and the non-uniformity of the compliance along the machining length decreases, the longitudinal accuracy of the shape of the part improves. In the case of rigid centers and a low-stiffness shaft, the compliance of the elastic technological system ω_1_(ε) is only the compliance of the shaft [38]:

(5) $$\omega_{1}(\varepsilon )=3.3\cdot 10^{-11}\cdot \varepsilon^{2}\cdot {\left(1-\varepsilon \right)}^{2}\cdot (K^{3}/d)$$

**Figure 2:**
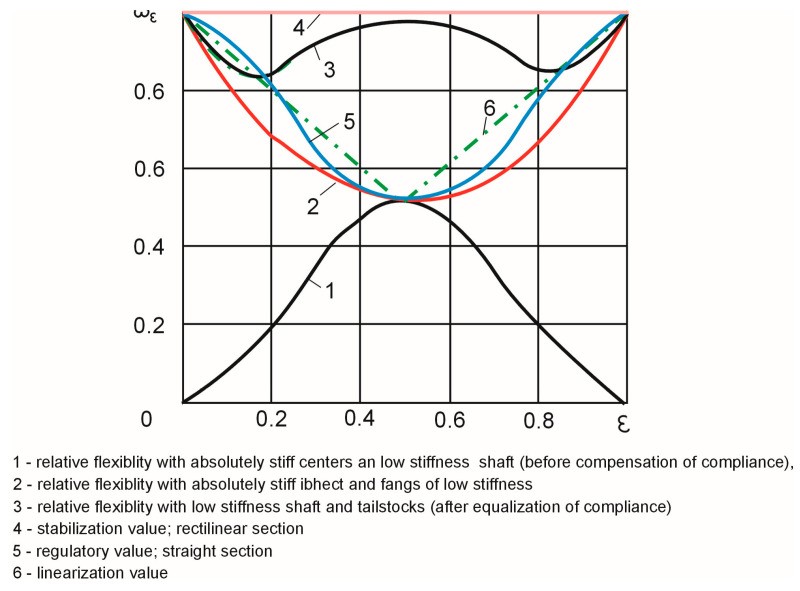
Relative compliance of elastic technological systems depending on the length of machining ϖ_ε_ = f(ε) [10].

This case corresponds to curve one in Figure 2.

For an absolutely rigid shaft and low-stiffness centers whose compliance is equalized to the value of ω_w_ = 4 × 10^−12^ ∙ K_r_^3/^d, the compliance of the elastic technological system is expressed by the equation [38]:

(6) $$\omega_{2}(\varepsilon )=0.4\cdot 10^{-11}\cdot [\varepsilon^{2}\cdot {\left(1-\varepsilon \right)}^{2}]\cdot (K^{3}/d),$$

which corresponds to curve two in Figure 1.

In a technological system consisting of a low-stiffness shaft and low-stiffness centers, the compliance of the system is the sum of the compliances of these elements, and is expressed by the relationship [38]:

(7) $$\begin{array}{c} \omega_{3}(\varepsilon )=\omega_{1}(\varepsilon )+\omega_{2}(\varepsilon )\\ \omega_{3}(\varepsilon )=3.3\cdot 10^{-11}\cdot \varepsilon^{2}\cdot {\left(1-\varepsilon \right)}^{2}\cdot (K^{3}/d)+0.4\cdot 10^{-11}\cdot [\varepsilon^{2}+{\left(1-\varepsilon \right)}^{2}]\cdot (K^{3}/d) \end{array}$$

Graphically, this case is represented by curve three in Figure 2, which is a graphical sum of the curves representing cases one and two. The curve illustrates the non-uniformity of compliance along the length of the treatment. This non-uniformity causes shape errors in the low-stiffness shafts.

Data on the non-uniformity of compliance and the relationships describing changes in non-uniformity of compliance over the length of machining allow one to assess the level of accuracy when compliance is passively evened out and to develop technological assumptions to further increase the accuracy of the shape of the low-stiffness shafts during grinding. When the relatively constant coefficients are normalized, Equations (5)–(7) take the following form [38]:

(8) $$\omega_{1}(\varepsilon )=8\cdot \varepsilon^{2}\cdot {\left(1-\varepsilon \right)}^{2}=8\cdot \varepsilon^{4}-16\cdot \varepsilon^{3}+8\cdot \varepsilon^{2},$$

(9) $$\omega_{2}(\varepsilon )=\varepsilon^{2}+{\left(1-\varepsilon \right)}^{2}=2\cdot \varepsilon^{2}-2\cdot \varepsilon +1$$

(10) $$\omega_{3}(\varepsilon )=8\cdot \varepsilon^{2}\cdot {\left(1-\varepsilon \right)}^{2}+\varepsilon^{2}+{\left(1-\varepsilon \right)}^{2}=8\cdot \varepsilon^{4}-16\cdot \varepsilon^{3}+10\cdot \varepsilon^{2}-2\cdot \varepsilon +1$$

The highest values of non-uniformity of compliance along the machining length, obtained by analyzing dependencies (8), (9), and (10) in terms of the maximum and minimum compliance values, are presented in Table 1.

An analysis of the data presented in Table 1 shows that, in the first and second case, the non-uniformity of compliance along the machining length is Δω_1,2_ = 0.5, and, in the third case (a low-stiffness shaft and low-stiffness centers), the non-uniformity of the compliance is Δω_3_ = 0.125. The relative non-uniformity of the technological system’s compliance after leveling out (increasing the compliance of the centers) is expressed by the ratio Δω_1,2/_Δω_3_ = 4. As can be seen, after the leveling out, the non-uniformity of the system’s compliance is four times smaller, and the shape error caused by the elastic deformations should, theoretically, also be four times smaller in this case.

The method of increasing the accuracy of the shape of parts by reducing the compliance of the centers is a passive one. A technological limitation of this method is that it requires the use of sets of centers of different stiffnesses and does not allow one to adjust the compliance during machining.

Given these drawbacks of the passive method, an active compliance compensation method for the elastic technological system used in the machining process was developed.

The characteristics illustrated in Figure 2 and the calculations provided in Table 2 show that a further increase in the machining accuracy is possible when the compliance is stabilized at the perpendicular level four (Figure 2), which corresponds to the maximum compliance value.

Stabilization can be achieved by adjusting the rigidity of the centers so that the elastic technological system’s compliance does not change according to curve two, but according to curve five (Figure 2). Then, curve five will represent the adjustment that ensures the stability of the compliance over the length of treatment ω_4_ = const. Curve five can be calculated using the following equation [38]:

(11) $$\omega_{5}(\varepsilon )=\omega_{4}(\varepsilon )-\omega_{1}(\varepsilon )=[0.4\cdot 10^{-11}-3.3\cdot 10^{-11}\cdot \varepsilon^{2}\cdot {\left(1-\varepsilon \right)}^{2}]\cdot (\kappa_{r}^{3}/d).$$

For the calculation and construction of curves, relation (11) can be transformed into an equation with constant coefficients [38]:

(12) $$\omega_{5}(\varepsilon )=1-8\cdot \varepsilon^{2}\cdot {\left(1-\varepsilon \right)}^{2}.$$

Curve five (Figure 2) and relationship (11), which describes it analytically, can be used to adjust the stiffness of the centers to a value that ensures the uniform compliance of the technological system and, at the same time, guarantees the greatest possible shape accuracy in the longitudinal section of the workpiece.

Curve two and its analytical expression (6) can also be the basis for adjustment. The difference between curve two and adjustment curve five in this case can be determined by the adjustment factor λ_ε_, which is given by the formula below [38]:

(13) $$\lambda \varepsilon =\frac{\omega_{5}(\varepsilon )}{\omega_{2}(\varepsilon )}=\frac{1-8\cdot \varepsilon^{2}\cdot {\left(1-\varepsilon \right)}^{2}}{\varepsilon^{2}+{\left(1-\varepsilon \right)}^{2}}.$$

**Figure 3:**
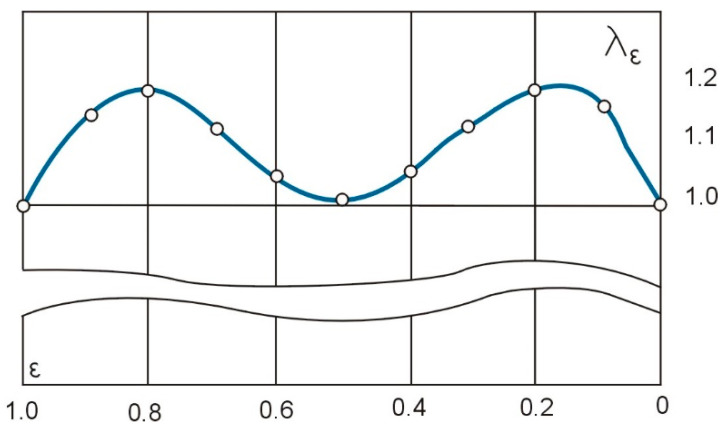
Curve of eccentric compliance adjustment over the machining length.

Formula (13) was used to obtain the adjustment values along the machining length shown numerically in Table 2 and graphically in Figure 3.

The adjustment made according to curve five is associated with practical difficulties. Therefore, it is better to approximate curve five to polyline six with two linear segments. As can be seen from the graph, line six has an inflection point located at ε = 0.5. For this coordinate, segments ε_I_ = 0–0.0 and ε_II_ = 0.5–1.0 of polyline six can be expressed by the following relationships [38]:

(14) $$\omega_{6}(\varepsilon_{I})=1-\varepsilon ,\;\omega_{6}(\varepsilon_{II})=\varepsilon .$$

The calculations of the relative deviation from compliance for the linearization of curve five (Table 2) show that the largest relative deviation is 10.1%, which is acceptable in practice. The relative deviation from compliance δ_ε_ is given by [38]:

(15) $$\delta (\varepsilon )=\frac{\Delta \omega_{6}}{\omega_{6}(\varepsilon )}\cdot 100\%,$$

where:

$\Delta \omega_{6}=\omega_{6}(\varepsilon )-\omega_{5}(\varepsilon )$—absolute deviation of curve 6.

When the rigidity is adjusted according to the linearized curve six, the changes in the compliance along the processing length can be determined from the relationship [38]:

(16) $$\omega_{7}(\varepsilon )=\omega_{1}(\varepsilon )+\omega_{6}(\varepsilon )$$

The non-uniformity of compliance is the largest from line four, with respect to the ordinate (16) and equals Δω_7_ (0.4; 0.6) = 0.061, which, theoretically, is 8.2 times less than the initial non-uniformity and 2 times less, when compared to the passive compliance compensation Δω_3_ = 0.125.

The results of the theoretical study show that the proposed method of increasing the accuracy of grinding of low-stiffness shafts, which consists of adjusting the compliance of the technological system, can be used in the machining of parts.

## 3. An Experimental Study on Increasing Machining Accuracy

### 3.1. General Characteristics of the Experimental Stand and the Experimental Procedure

During the machining of low-stiffness machine elements, there arise complex relationships and functional dependencies which are characteristic of the specific types and conditions of machining. Any theoretical assumptions about the machining of this kind of parts should therefore be confirmed by experimental studies. Experimentally established relationships and dependencies allow one to more accurately predict errors, rationally select machining parameters, and control part deformation.

The present experimental tests were carried out to check the theoretical assumptions for controlling the grinding accuracy of low-stiffness shafts.

The tests were performed on a 3B153 cylindrical grinder (produced by the grinder factory in Vilnius). A ceramic bond grinding wheel made of aluminum oxide with the dimensions of 400×40×127, grain size 40, and hardness SM1 was used. The measurement of the force components was carried out using a dynamometer type 9123 by Kistle.

Calibrated rollers made of C50 steel (C: 0.47–0.55, Si: 0.17–0.37, Mn: 0.5–0.8, S < 0.04, P < 0.035, Cr < 0.25) were used as specimens, which allowed us to exclude the influence of the input shape inaccuracy on the results of the experiment. The dimensions of the samples were selected based on the condition of equal stiffness at L/d = 20. Two types of samples were used: (1) length: 280 mm, diameter: 6 mm; (2) length 280 mm, diameter: 6 mm. Each sample was ground in 12 duplicate passes. The experiments were carried out under the following grinding conditions: cross-feed a_p_ = 0.01 mm/double pass, longitudinal feed f = 16 mm/rev, cutting speed v_c_ = 12 m/min.

The grinding force was calculated using the experimental relationship (17), depending on the dimensions of the samples, the grinding parameters, and the coordinates of the position of the grinding wheel relative to the machined surface. To increase the reliability of the experiment, each trial was repeated six times:

(17) $$F_{p}=3.6+78,1\cdot f_{2}-0.23\cdot f_{1}-0.39\cdot V_{d}-0.04\cdot d+4.45\cdot \varepsilon +0.03\cdot f_{1}\cdot V_{d}-5.9\cdot \varepsilon^{2}$$

The average value of the calculated stabilized resistive force was *F_p_* = 15 N. In the experiments, standard centers with a diameter of 22 mm were used. To adjust the compliance, the cross-section of the *d* = 22 mm standard centers was reduced using a special groove, and the dimensions of this cross-section varied depending on the dimensions and compliance of the test samples. For shafts with a diameter of 8 mm, the flexibility of the centers should be equal to 4.2 µm/N, and for shafts with a diameter of 14 mm, the flexibility of the centers should be equal to 2.3 µm/N. In practice, the use of the grooves reduced the compliance of the centers to 4 µm/N when the exposed length of the centers was 85 mm, and to 2 μm/N when the exposed length of the centers was 70 mm.

The experiments allowed us to determine whether it was possible, in practice, to increase the machining accuracy by controlling the compliance and bending moment.

The initial experimental studies were conducted under the following conditions:

− the stiffness of the centers significantly exceeded the stiffness of the semi-finished product (compliance was not leveled out or adjusted);
− the compliance of the centers was double the compliance of the semi-finished product and did not change throughout the machining process (passive compliance compensation);
− the centers’ compliance was adjusted along the machined surface;
− the value of bending moments applied to the ends of the workpiece changed with the position of the grinding wheel along the machining length.

Experimental data on the maximum shape deviations of the shafts (Table 3) show that the most effective methods for increasing the grinding accuracy were adjusting the compliance and controlling the bending moments depending on the position of the cutting force (grinding wheel) along the machining length.

### 3.2. Abrasive Machining of Low-Stiffness Shafts with Adjustment of Center Compliance

Taking into account the fact that the passive compliance compensation does not permit one to significantly increase the machining accuracy, a method for the active compensation of the compliance of an elastic technological system during the machining process was developed.

The calculations (Table 2 and Figure 2) show that the machining accuracy can be enhanced by stabilizing the system’s compliance at the level of perpendicular 4 (Figure 2), which corresponds to the maximum compliance value.

**Figure 4:**
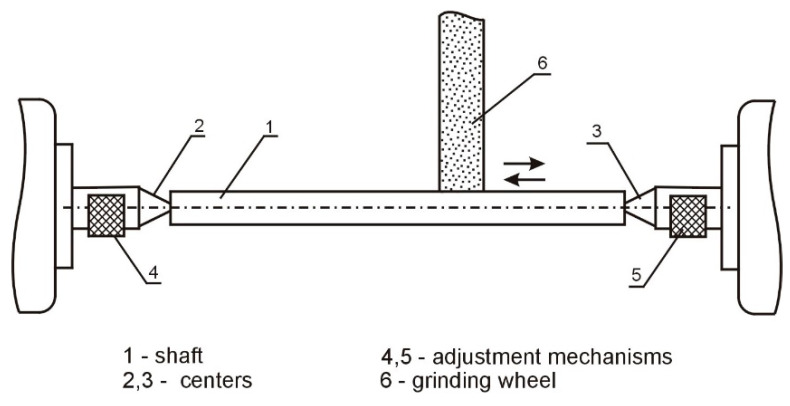
Schematic of the method of grinding low-stiffness shafts with an adjustment of compliance.

A schematic of the method for grinding low-stiffness shafts in centers, with an adjustment of compliance, is shown in Figure 4.

The method consists of adjusting the stiffness of the centers 2 and 3 during the machining process, depending on the position of the grinding wheel 6 along the shaft 1, by means of adjustment mechanisms 4 and 5, located in the shanks of centers 2 and 3.

**Figure 5:**
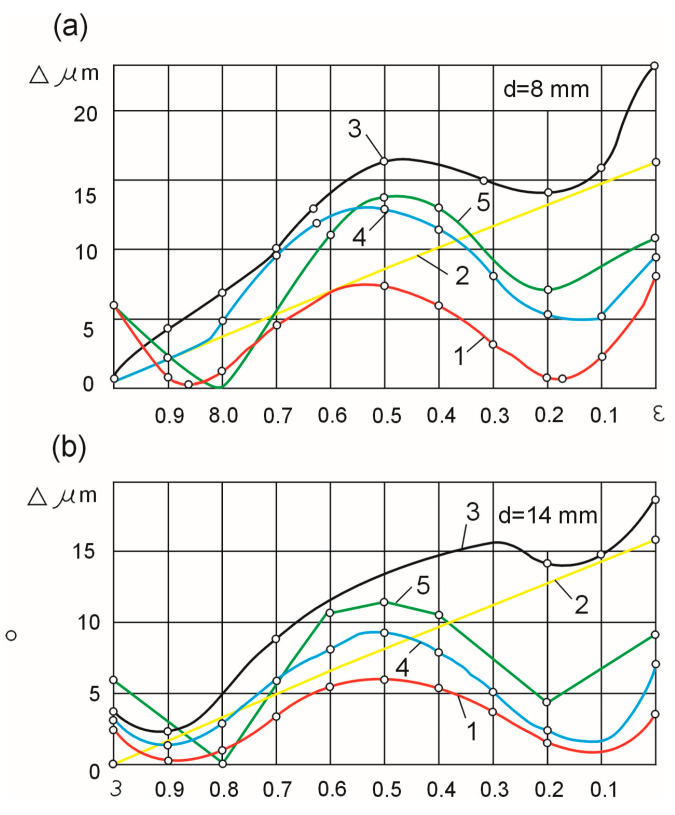
Shaft cylindricity errors when regulating the compliance of the investigated technological system (horizontal axis ε = x/L): (**a**) for d = 8 mm, (**b**) for d = 14 mm.

When the compliance is adjusted by a targeted increase in the compliance of the centers and there are no geometrical inaccuracies of the machine tool, the compliance of the two centers should be identical: for samples with diameters d = 8 mm and d = 14 mm, 4 µm/ N and 2 μm/N, respectively. Curve 1 in Figure 5 shows the theoretical deviation of the shape of the workpiece when the compliance of the technological system is adjusted.

The geometrical inaccuracy of the machine tool determined from the dependence (17) (curve 2) introduces a systematic error. The theoretical deviation of the shape is represented by curve 3 of Figure 5. The largest shape deviation equals 23 μm for shafts with a diameter of d = 8 mm, and 19 μm for shafts with d = 14 mm. These values are close to the experimental shape deviation values for rigid centers (32 µm and 22 µm, respectively). This means that when the machine tool has geometrical inaccuracies, the uniform compensation of the compliance of the technological system does not increase the shape accuracy of the workpiece.

In order to compensate for the initial displacement of the workpiece axis, the compliances of the headstock and the tailstock were set as unequal during the experiments. For samples when d = 8 mm, the compliance of the headstock was assumed to be 4 µm/N and the tailstock’s compliance was assumed to be 3 µm/N. For samples when d = 14 mm, the respective compliances were set to 2 µm/N and 1 µm/N. Curve 4 of Figure 5 shows the theoretical deviation of the shape of the samples for these compliance values when the machine tool shows geometric inaccuracies. An analysis of curves 4 and 3 leads to the conclusion that the method of unequal weakening (reduction in the compliance) of the centers makes it possible to compensate for the deviation of the shape resulting from the geometric inaccuracies of the machine tool and allows for increasing the machining accuracy.

The results of the experimental tests, shown as curve 5 of Figure 5, indicate that such an approach is sound. With such determination, taking into account the compensation of the geometrical inaccuracy of the headstock and the tailstock, which varies depending on the length of the shaft, the largest experimental shape deviation was obtained, equal to 14.5 µm for shafts with d = 8 mm and 11.5 µm for shafts with d = 14 mm. The values of shape deviations were half of those obtained for a set-up with evenly weakened centers.

The dispersion analysis (Table 4 shows the calculations for shafts with d = 8 mm) indicates that, at the confidence interval P = 0.95, the experimental results are lower than those in the tables (G_o_ < G_T_); the difference between the theoretical and the experimental values of the mean shape deviation is non-significant (t_p_ < t_t_; t_p_* < t_t_*), and the experimental results are consistent with the real process (F_p_ < F_T_).

The experiments were provided to test the effects of the linearization adjustment of compliance according to the dependence (14), and programmed adjustment of compliance according to dependence (11).

For parts with d = 8 mm, the linearization adjustment of the compliance along the length of the workpiece yielded a compliance value of 3 m/N at ε=0. For the linear movement of the grinding wheel along the workpiece, the compliance value was reduced to 2 m/N at ε = 0.5 and increased to 4 µm/N at ε = 1.0. Similarly, for samples with d = 14 mm, the compliance decreased from 1.5 µm/N to 1.0 µm/N at ε = 0, and then increased to 2 µm/N at ε = 1.0. The programmed adjustment of the compliance of the technological system in accordance with (11) is shown as the theoretical adjustment curve 6 (Figure 2). Such an adjustment should theoretically ensure zero shape deviations.

The experiments show that, compared with passive compliance adjustment, the linearization adjustment leads to a two-fold increase in the shape accuracy, while the programmed adjustment may result in even a four-fold increase in accuracy. When compared to the uncontrolled grinding of low-stiffness shafts with d = 8 mm and d = 14 mm, the programmed adjustment of compliance increased the shape accuracy by one order of magnitude.

### 3.3. Abrasive Machining of Low-Stiffness Shafts with Control of Bending Moments

**Figure 6:**
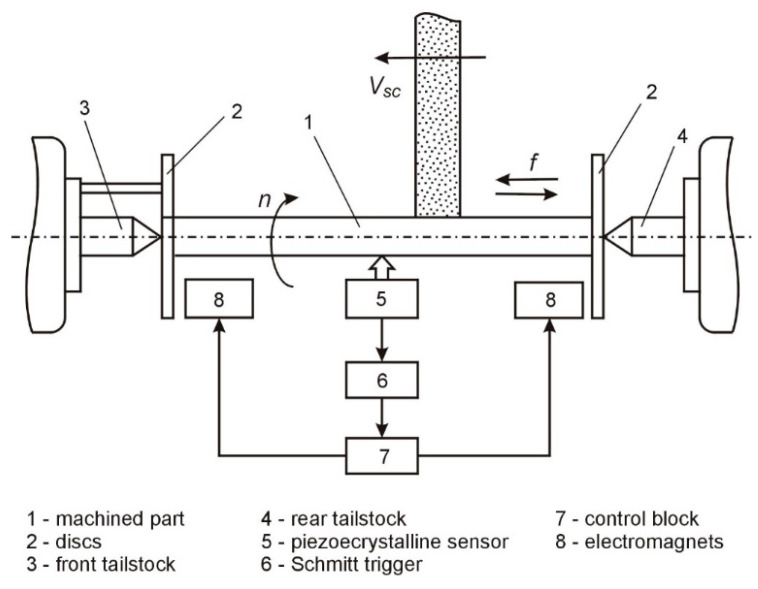
Block diagram of the measurement and compensation system of bending vibrations during grinding [10].

The abrasive machining of low-stiffness shafts was carried out on a station which allows for the controlling of bending moments. A block diagram of the station is shown in Figure 6, and the operational characteristics of the station are presented in [27].

When controlling the shape accuracy of a workpiece by changing the bending control moments, the values of those moments are determined along the machining length.

The following input data were adopted: actual grinding force *F_y_* = 15 N; the ratio of the length of the sample to its diameter L/d = 20; the (exposed) length of the centers a_1_ = 50 mm; the current coordinate of the position of the cutting force x = 0, L/4, L/2, 3 L/4, L. The relative displacement of the left end of the workpiece was taken to be 1 (ν = 1), and the machine tool’s geometric inaccuracy (displacement: on the grinding wheel of the headstock) was conventionally transferred to the right stop (f = ∆l/∆p)–the initial displacement of the left and the right end of the workpiece, respectively.

For samples with d = 8 mm and L = 160 mm (L/d = 20 mm), the ratio of the moments of inertia of the centers (I2) and the workpiece (I1) was assumed to be n = I2/I1 = 4 (standard centers with a diameter of 31 mm were used); the ratio a1 of the length of the supporting center to length L of the workpiece m = a/L = 0.31. For samples with d = 14 mm and a length of 280 mm (L/d = 20), n = 2.5 and m = 0.18.

**Figure 7:**
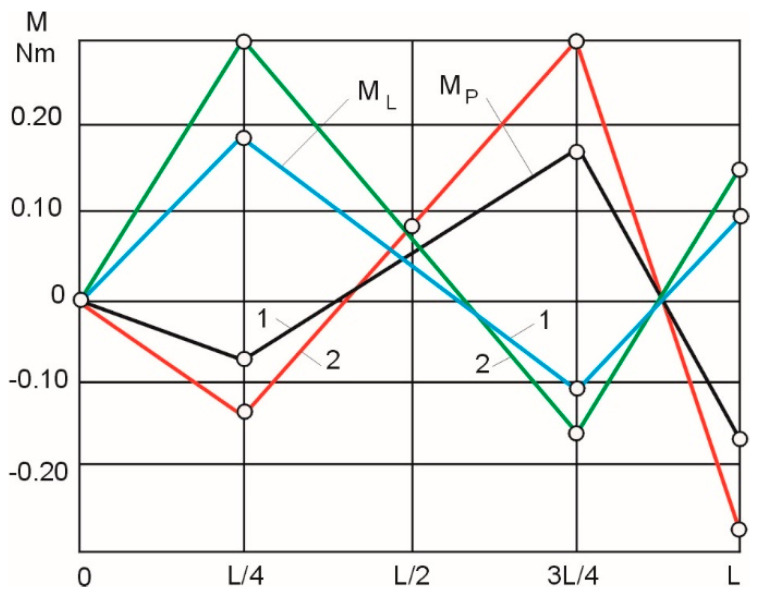
Linearized values of the control moments on the left (Ml) and right (Mp) stops: 1—for samples with d = 8 mm; 2—for samples with d = 14 mm.

The calculated values of the right (M_p_) and left (M_l_) bending moments are shown in Figure 7 in a linearized form (curve 1—for samples with a diameter of 8 mm, curve 2—for samples with a diameter of 14 mm). The values of the bending moments were linearized to simplify the control programs used in the test device.

**Figure 8:**
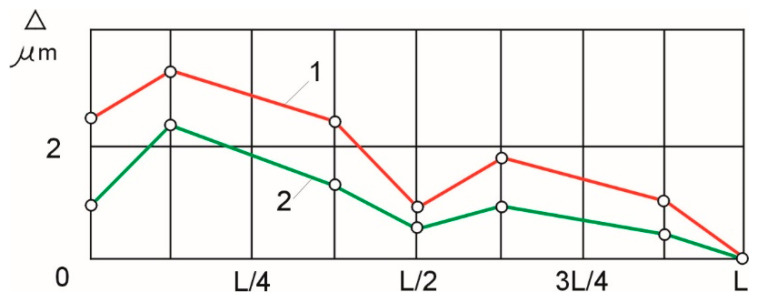
Shape deviations of samples d = 8 mm (1) and d = 14 mm (2), when bending moments were controlled.

When the control of the bending moments was linearized in accordance with Figure 7, the actual experimental shape deviations of the samples with a diameter of 8 mm (curve 1) and 14 mm (curve 2) were as shown in Figure 8.

The dispersion analysis shows that the results of the experiments are repeatable (G_p_ < G_r_), and the deviations of the mean values of the experimental curves 1 and 2 (Figure 7), when the control of the bending moments is linearized, are non-significant (t_p_ < t_r_).

The largest shape deviations (3.3 µm for shafts with d = 8 mm and 2.1 µm for shafts with d = 14 mm) are within the range of the tolerance zone, according to accuracy class 6. These shape accuracy values are an order of magnitude greater than those obtained in the uncontrolled process.

The experimental studies confirm the theoretical assumption that technological methods can be used to obtain quite a large increase in the accuracy of the shape of low-stiffness shafts produced by grinding. In the present study, the accuracy was increased by controlling the technological system’s displacement directly during the machining process.

The experiments we carried out show (Figure 8) that the shape deviations of the samples can be reduced by an order of magnitude when bending moments are controlled, compared to the uncontrolled process.

## 4. Conclusions

This paper proposes a method of increasing the workpiece’s shape accuracy by controlling the compliance of the technological system. This provided study allows one to draw the following conclusions:

− In grinding operations, shape errors in the longitudinal section of the shaft are mainly caused by the uneven stiffness of the technological system at the point of the application of the cutting force. They can be reduced by equalizing the stiffness of the technological system at the cross-sections of the workpiece (in places where the cutting force is applied).
− The accuracy of the shape of a shaft can be increased up to four times by reducing the compliance of the centers (passive method). However, this approach requires the use of sets of centers with different stiffness values and it does not allow for the adjusting of the compliance of the system during machining.
− These limitations of passive compliance compensation have been eliminated in the proposed method of active compensation of the compliance of the elastic technological system during the machining process. In the active approach, the compliance is stabilized by adjusting the stiffness of the centers.
− When the compliance is regulated by a targeted increase in the compliance of the centers, and there are no geometrical inaccuracies of the machine tool introducing a systematic error in the machining results, the compliance of the two centers should be the same. However, when the grinding machine has geometrical inaccuracies, the uniform compensation of the technological system’s compliance does not increase the shape accuracy of the workpiece.
− Control of bending moments allows for the reduction of the shape deviations of the samples by one order of magnitude compared to the uncontrolled process.

## Figures and Tables

**Table 1:** Values of non-uniformity of compliance of the technological system along the machining length.

The Ordinal Number of the Equation	Relative-Compliance Values at Experimental Points		Compliance Values at Experimental Points		Non-Uniformity of Compliance
	Ɛ_max_	Ɛ_min_	ω_max_	ω_min_	Δω
(9)	0.5	1.0	0.5	0	0.5
(10)	1.0	0.5	1.0	0.5	0.5
(11)	0 0.5 1.0	0.15 0.85	1.0	0.875	0.125

**Table 2:** Results of calculations of the relative compliance of the elastic technological system.

Reference Coordinate	Compliance							Regulation Factor *λƐ*	Absolute Deviation Δ*ω*_6_	Relative Deviation *δ(Ɛ)%*	Non-Uniformity of Compliance Δ*ω*_7_
	*ω*_1_ *(* *Ɛ)*	*ω*_2_ *(* *Ɛ)*	*ω*_3_ *(* *Ɛ)*	*ω*_4_ *(* *Ɛ)*	*ω*_5_ *(* *Ɛ)*	*ω*_6_ *(* *Ɛ)*	*ω*_7_ *(* *Ɛ)*				
0	0	1.0	1.0	1.0	1.0	1.0	1.0	1.0	0	0	0
0.1	0.065	0.82	0.885	1.0	0.935	0.9	0.965	1.14	0.035	3.7	0.035
0.15	0.132	0.74	0.875	1.0	0.868	0.85	0.982	1.173	0.018	2.1	0.018
0.2	0.205	0.68	0.885	1.0	0.795	7	1.005	1.169	0.005	0.6	0.005
0.3	0353	0.58	0.933	1.0	0.647	0.5	1.053	1.115	0.053	7.5	0.053
0.4	0.461	0.52	0.981	1.0	0.539	0.6	1.061	1.036	0.061	10.1	0.061
0.5	0.5	0.5	1.0	1.0	0.5	0.5	1.0	1.0	0	0	0
0.6	0.641	0.52	0.981	1.0	0.539	0.6	1.061	1.036	0.061	10.1	0.061
0.7	0.353	0.58	0.933	1.0	0.647	0.7	1.053	1.115	0.053	7.5	0.053
0.8	0.205	0.68	0.885	1.0	0.795	0.8	0.005	0.169	0.005	0.6	0.005
0.85	0.132	0.74	0.875	1.0	0.868	0.85	0.982	1.173	0.018	2.1	0.018
0.9	0.065	0.85	0.885	1.0	0.935	0.9	0.965	1.14	0.035	3.7	0.035
1.0	0	1.0	1.0	1.0	1.0	1.0	1.0	1.0	00	0	0

**Table 3:** Experimental values of maximum shape deviations (cylindricality) of low-stiffness shafts machined using different control methods.

No.	Control Methods	Cylindrical Deviation [µm] for Shaft Diameters	
		8 [mm]	14 [mm]
1.	No control	32.0	22.0
2.	Uniform compliance compensation	23.0	19.0
3.	Non-uniform compliance compensation	14.5	11.5
4.	Linearizing adjustment of compliance	7.0	4.5
5.	Programmed compliance adjustment	3.5	2.5
6.	Control of bending moments (linearization)	3.3	2.1

**Table 4:** Dispersive analysis of the results of experimental tests of samples with diameter d = 8 mm with non-uniform compensation of the compliance of the technological system.

Relative Coordinate ε	Ordinates of Curves Xi		Deviations of Ordinates ΔX_i_		Dispersion of Deviations S_i_^2^	
	(4) Theoretical X_1_	(5) Experimental X_2_	$\left(X_{1}-{\bar{X}}_{1}\right)$	$\left(X_{2}-{\bar{X}}_{2}\right)$	${\left(X_{1}-{\bar{X}}_{1}\right)}^{2}$	${\left(X_{2}-{\bar{X}}_{2}\right)}^{2}$
0	9.0	11.5	1.6	3.2	2.56	10.24
0.1	3.6	9.1	3.8	0.8	14.44	0.64
0.2	4.7	7.0	2.7	1.3	7.29	1.69
0.3	8.4	10.5	1.0	2.2	1.0	4.84
0.4	11.6	14.0	4.2	5.7	17.64	32.49
0.5	13.2	14.5	5.8	6.2	33.64	38.44
0.6	12.0	10.5	4.6	2.2	21.16	4.84
0.7	8.8	5.0	1.4	3.3	1.96	10.89
0.8	5.3	0	2.1	8.3	4.41	68.89
0.9	4.3	3.0	3.1	5.3	9.61	28.09
1.0	0	6.0	7.4	2.3	54.76	5.29
∑	80.9	91.1	-	-	168.47	206.34
${\bar{X}}_{i}$	7.4	8.3	-	-	-	-
S_i_^2^	$S_{i}^{2}=\frac{\sum_{i=1}^{n}{\left(X_{i}-{\bar{X}}_{i}\right)}^{2}}{n-1}$ $S_{i}=\sqrt{S_{i}^{2}}$ $G_{i}=\frac{S_{\max}^{2}}{\sum S_{i}^{2}}$ $t_{i}=\frac{{\bar{X}}_{i}}{S_{i}}$ $t=\frac{\left|{\bar{X}}_{2}-{\bar{X}}_{1,3}\right|}{\sqrt{n_{1}\cdot S_{1,3}+n_{2}\cdot S_{2}^{2}}}\cdot \sqrt{\frac{n_{1}\cdot n_{2}\cdot (n_{1}+n_{2}+2)}{n_{1}+n_{2}}}$ $F=\frac{S_{2}^{2}}{S_{1,3}^{2}}$				S_1_^2^ = 16.58	S_2_^2^ = 20.63
S_i_					S_1_ = 4.1	S_2_ = 4.5
G_i_ = 0.349					G_1_ = 0.32	G_2_ = 0.33
t_T_ = 2.2					t_1_ = 1.8	t_2_ = 1.84
t*_T_ = 2.08					t*_1–2_ = 0.47	
F_T_ = 3.59					F_1–2_ = 1.22	

## Data Availability

Not applicable.
